# Piercing and sucking mouth parts sensilla of irradiated mosquito, *Culex pipiens* (Diptera: Culicidae) with gamma radiation

**DOI:** 10.1038/s41598-022-22348-0

**Published:** 2022-10-25

**Authors:** Nagwan Zahran, Sameh Sawires, Ali Hamza

**Affiliations:** Department of Natural Products Research, National Center for Radiation Research and Technology (NCRRT), Egyptian Atomic Energy Authority (EAEA), Cairo, Egypt

**Keywords:** Zoology, Diseases

## Abstract

Morphology, distribution and function of the sensilla associated with mouthparts of female mosquito, *Culex pipiens* (Diptera: Culicidae) were studied by using the scanning electron microscope. The female mosquito mouthpart is a piercing and sucking type and carrying many sensory organs which plays major role in host seeking, and oviposition behaviour of mosquitoes. Six types of mouthpart sensilla have been identified and described, Sensilla trichoidea, chaetica, basiconica, Capitate peg, campaniformia and squamiformia. Females mosquito were irradiated as pupae with 20, 40 and 80 Gy of gamma radiation to investigate their effect on mouthparts sensilla. Dose of 20 Gy had slight effect on the different types of the sensilla as compared with the control. Features of malformation observed in the maxillary palp segments at this radiation dose include falling of some scales (sensilla squamiformia) at certain parts of the 1st segment leaving vacuoles. At 40 Gy, the maxillary palp segments were liquefied in some areas leaving undistinguished shape. At the highest dose 80 Gy, the tip of labial palps had many tears and showed were most affected, especially, at the trichoid, basiconic and chateica sensilla. All these malformations may lead the mosquitoes to not reaching the host, impeding their ability to transmit diseases or killing the mosquitoes, and this is the main objective of this study.

## Introduction

*Culex* is a mosquito genus which is widespread in tropical, subtropical and temperate climates, both in urban and rural environments around the world^1^. *Culex* mosquitoes, especially *Culex pipiens* and *Cx. quinquefasciatus*, are the main vectors of filariasis in many regions of the world including the Middle East and eastern Mediterranean countries^2^.

Mosquito mouthparts are structurally adapted for the uptake of fluid. Those of the blood-sucking females are highly specialized for piercing skins and sucking blood^3,4^. Their mouthpart, the proboscis, is formed by a fascicle that consists of six stylets which is the same length as the labium. During blood feeding, the fascicle enters the skin, whereas the labium remains on the skin. Unlike females, male mosquitoes do not feed on blood and their maxillae and mandibles are much shorter than the proboscis. The food of male mosquitoes is floral and extra floral nectar, honeydew^5^ or even plant tissue^6^. In blood-sucking females, the toothed maxillae function as hooks and anchor the fascicle to the hosts’ skin^4^. It is not clear whether the mandibles play an active role during piercing as in other blood-sucking nematoceran flies^3^ or simply serve as a valve regulating the size of the distal opening of the labral food canal^4^.

The sensory mechanism plays a significant role in host-seeking and oviposition behaviour of mosquitoes, which enable them to transmit various diseases to humans^7^. The sensilla are sensory recievers located at peculiar locations in the body of insects such as the antennae, maxillary palps, proboscis, tarsi and tergum. The sensilla exist in several forms. Each is specialized to receive a well-defined stimulus such as mechanical effects, temperature or humidity changes, or any kind of odors^8^. The behavioral responses of female mosquitoes to find their host are a vital factor in their potential for disease transmission. Bohbot et al.^9^ studied maxillary palp sensilla of *Aedes aegypti* and its potential involvement with sensory modalities. Dhanalakshmi et al.^10^ reported that female mosquito found its hosts by produced carbon dioxide.

Many authors had studied the effect of gamma radiation on the sensilla of different insects. Haiba^11^ on the legs of potato tuber moth, *Phthorimae operculellaea*; El-Akhdar^12^ on wings and mouth parts of the Mediterranean fruit fly, *Ceratitis capitata*; Hazaa^13^ on antennal sensilla in the male moth of the cotton leaf worm, *Spodoptera littoralis* and Zahran^14^ on ovipositor sensilla of the peach fruit fly, *Bactrocera zonata*. These authors found many of malformations and changes in the shape of the sense organs as a result of the effect of gamma radiation. No references have previously shown the effect of gamma radiation on the mouthparts of a *Cx. pipiens* female and their sensilla. So, the main purpose of this study is to describe the types and distribution of sense organs on the mouthparts of female *Cx. pipiens* using scanning electron microscopy. Furthermore, the effect of gamma radiation with different dose levels (20, 40 and 80 Gy) on the morphological structure of mouthpart and sensilla and the possible effect of gamma radiation on the function of these sinsilla were investigated.

## Materials and methods

### Mosquito colony

Mosquito eggs and larvae were collected from suitable breeding sites such as water ponds around Cairo and Al-Qualyobia Governorates during June and July 2020. The samples were transferred to the laboratories of the Entomology Unit, Nuclear Research Center, Egyptian Atomic Energy Authority (EAEA), Cairo, Egypt where self-perpetuating colonies were established and maintained during the present study. Mosquitoes were reared for many generations before performing experiments under controlled laboratory conditions (27 ± 2 _C, 70 ± 10% RH and 12–12 light–dark regime) according to the method described by^15^.The eggs were put into metal plates containing dechlorinated tap water and were reared until the 3rd instar larvae. Larvae were fed a diet of grounded fish food and raised until they grew into pupae and then transferred it's to the gamma irradiation unit.

### Irradiation technique

Newly emerged pupae were irradiated with low as 20 Gy, moderate as 40 Gy and high as 80 Gy with dose rate of 1.25 rad/s. using Gamma cell-40 (cesium-137) irradiator unit located at the National Center for Radiation Research and Technology (NCRRT), Nasr City, Cairo, Egypt. These doses were used based on a previous study by Ibrahim et al.^16^ it was tested on the antennae of female mosquitoes in which the newly emerged pupae were exposed to gamma doses and when the adults appeared the adult females were separated. The non-irradiated female pupae have been prepared in the same laboratory under the same conditions.

### Scanning electron microscopy

For the separation of females from males, the females have a more needle-like proboscis, which they use for biting. Males have bushy, hairy antennae, while the antennae of females are a lot less hairy. The head capsule of the female mosquitos were carefully cut by means of a sharp razor blade; the specimens were quickly dried then mounted on specimen stubs with gold conducting paints. Samples were gold coated in a layer of approximately 300 A°, using a fine gold coating apparatus, ion sputtering device (JEOL-JFC-1100E).

Examinations of mosquito mouthparts were carried out by a JOELJSM-5400 scanning electron microscope (SEM) with an accelerating voltage of 30 kV. The mouthparts sensilla were viewed and photographed directly from the SEM video monitor.

## Results

### Female mosquito, *Culex pipiens* mouthparts and the associated sense organs

The type of the mouthparts in female mosquito, *Cx. pipiens* is piercing sucking that carries various sensory organs that are responsible for the behavior of the insect and its response to surrounding environmental stimuli. The results of the present work reported by a simple observation of the different types of mouthparts sensilla detected on female mosquitos. Subsequently, changes induced in the sensilla of female adults irradiated as pupae with (20, 40 or 80 Gy) were reported.

**Figure 1:**
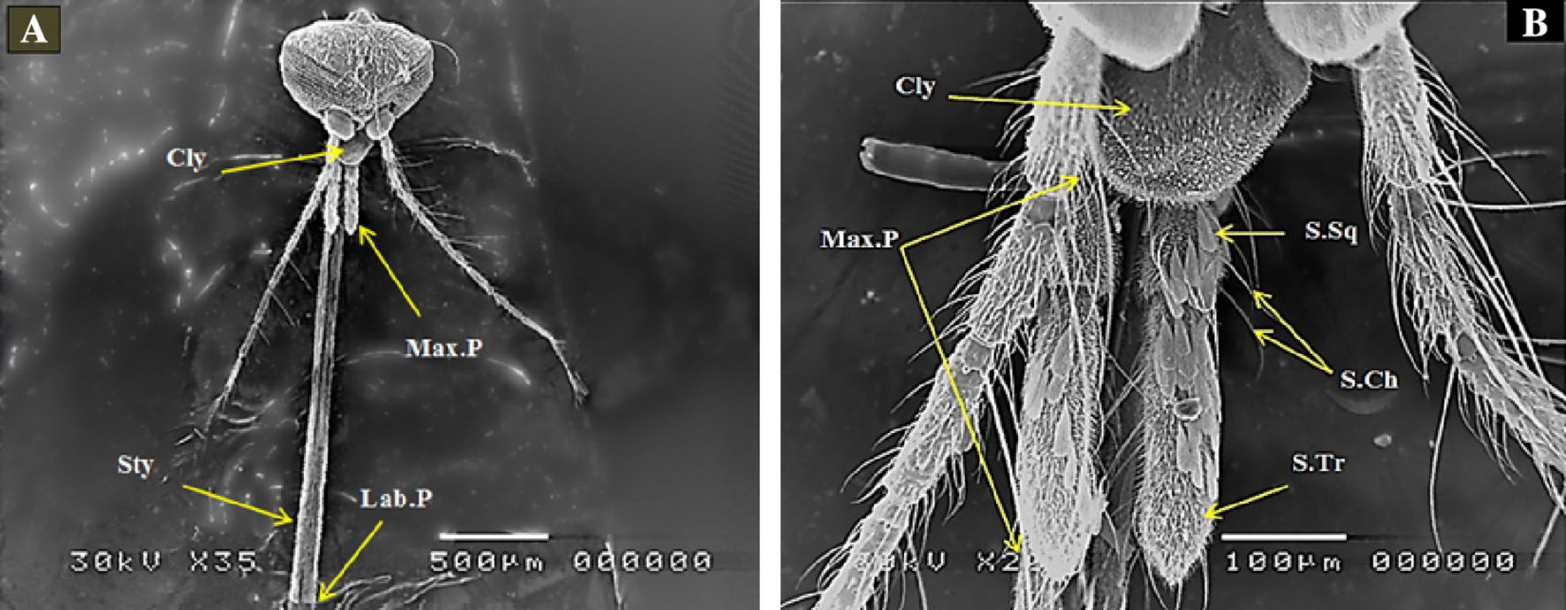
SEM photography of control female mosquito, *Culex pipiens* mouthparts (35 and 250×) showing (**A**) Clypeus (Cly), Stylets (Sty), Maxillary Palps (Max.P) and Labial Palps (Lab.P), (**B**) showing different types of sensilla; scales squamiformia (S.Sq), chaetica (S.Ch) and trichodea (S.Tr).

The female proboscis consists of 6 slender stylets (1 labrum, 2 mandibles, 2 maxillae and 1 hypopharynx which lie in the labium groove), 2 maxillary palps (each containing 4 segments) and 2 labial palps (Fig. 1A,B).

Mandibles are long and sharply pointed for piercing the host’s skin. The sucking tube consists of the labrum in anterior side and mandibles in posterior side. Food is drawn up the food channel which is a groove on the posterior side of the labrum. The hypopharynx contains the salivary duct and the labium is a large, thick appendage with a deep anterior groove.

### The types of sensilla on the mouthparts of the female mosquito, *Culex pipiens*

Various types of mouthparts sensilla have been identified and described by the scanning electron microscopic. On the basis of size, shape and structural features; many types of hair-like structures were noted as follows.

### Sensilla trichoidea (S.Tr)

**Figure 2:**
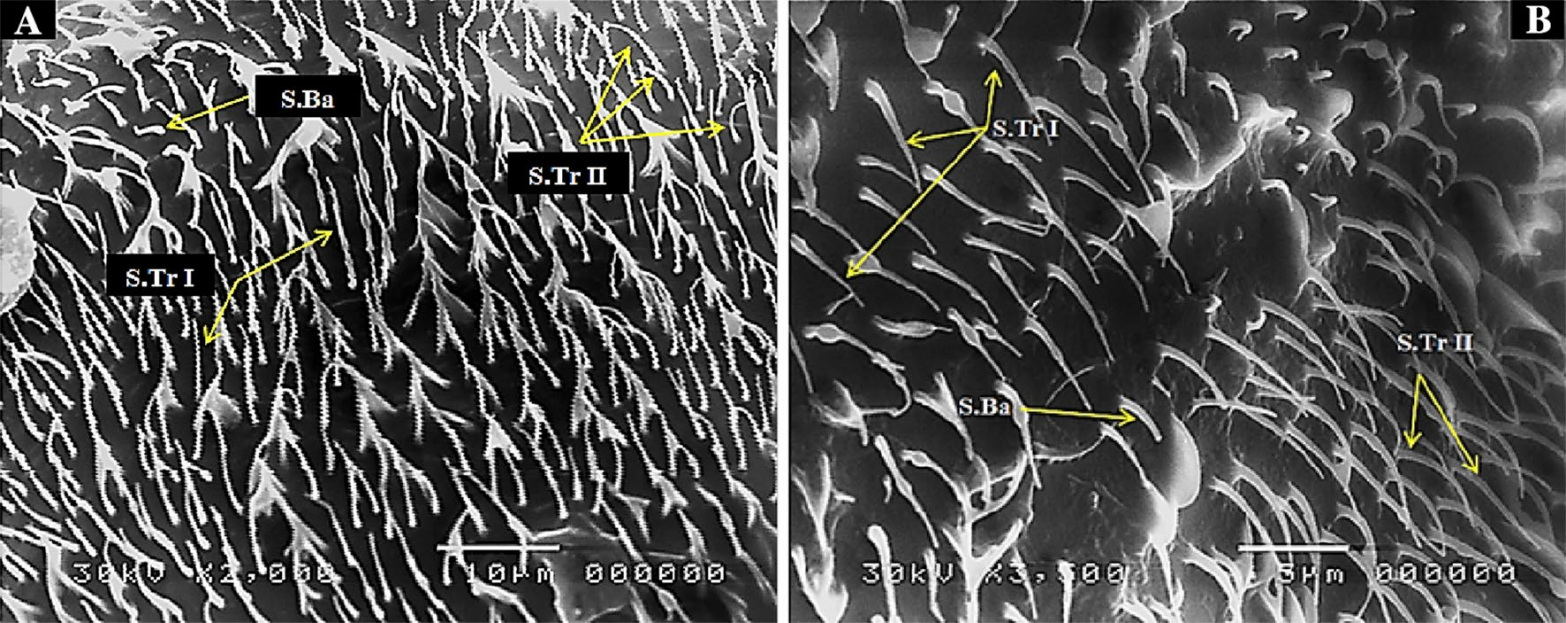
SEM photography of control, *Culex pipiens* female (2000 and 3500×) (**A**, **B**) showing clypeus covered heavily with the sensilla trichodea (S.Tr I& S.Tr II) and sensilla basiconica (S.Ba).

It is straight hair, tapering to a fine end, without openings or pores and growing out from a socket in the cuticle. These sensilla are chemosensitive, olfactory organs or thermosensitive^17^. This type of sensilla is the most abundant sensilla on clypeus and maxillary palps which are pointed or blunt. They are classified into following types based on their morphology: short sharp tipped trichodea (S.Tr I), short curved with a blunt tip (S.Tr II) and short sharp hooked at the tip (S.Tr III) (Figs. 2, 4).

### Sensilla chaetica (S.Ch)

**Figure 3:**
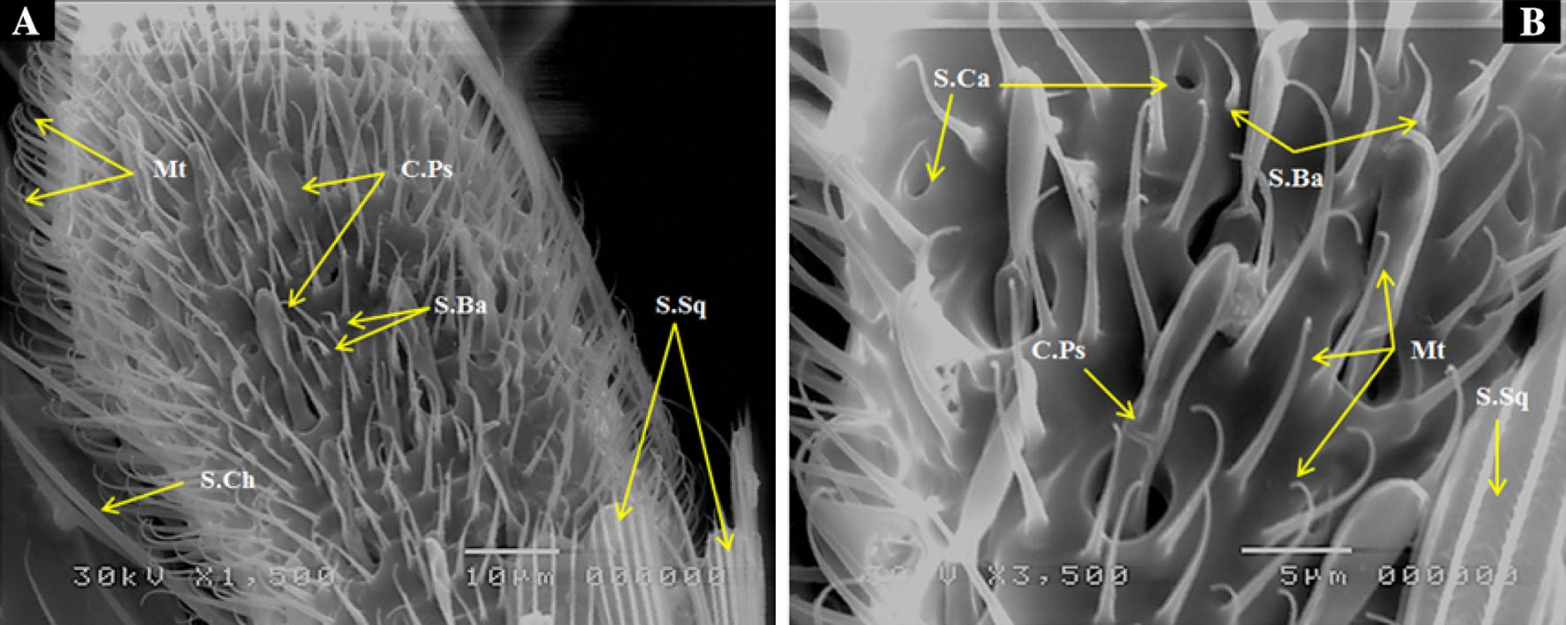
SEM photography of control, *Culex pipiens* female 4th distal end of maxillary palp (1500 and 3500×) showing (**A**) densely covered with the microtrichia (Mt), individual olfactory capitate peg sensilla (C.Ps), sensilla basiconica (S.Ba), sensilla chaetica (S.Ch) and sensilla squamiformia (S.Sq). (**B**) Showing stress sense organs sensilla campaniformia (S.Ca) at higher magnification.

**Figure 4:**
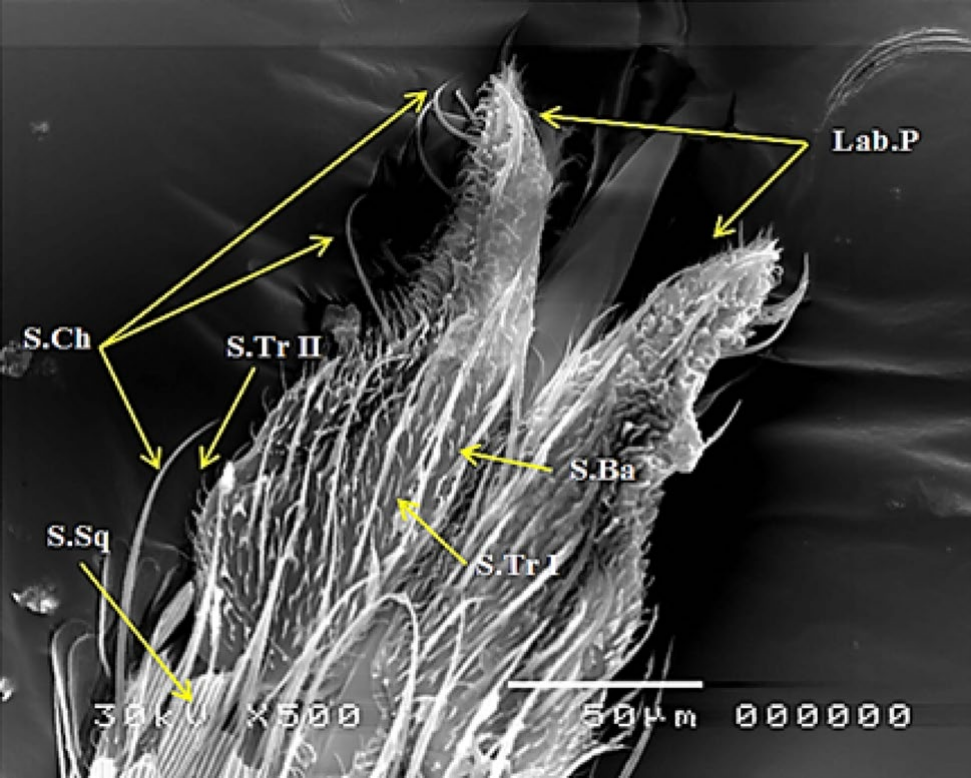
SEM photography of control, *Culex pipiens* female labial palps (500×) showing three size of smooth sensilla chateica (S.Ch) at the tip of labellum, sensilla trichodea (S.Tr I& S.Tr II), sensilla basiconica (S.Ba) and sensilla squamiformia (S.Sq).

This type is the most common sense organs located on the maxillary palp and they are long hair-like structures (Figs. 3, 4). They are similar in shape to sensilla trichodea (S.Tr I), but much longer in length and have a function as mechano-sensilla or tactile and perhaps chemosensitive.

### Sensilla basiconica (S.Ba)

They were cone-shaped sensilla with a blunt tip and a smooth surface; they were located among sensilla trichodea and act as hygrosensitivity. This type of sensilla is found in clypeus and maxillary palp. (Figs. 2, 3, 4).

### Capitate peg sensilla (C.Ps)

These are the pegs, club-shaped sensilla broadened at the tip and arising from a circular depression. These pegs were found at the distal end of maxillary palp and are possibly olfactory in function (Fig. 3). These sensilla are thin walled and occur on the 4th segment of maxillary palp.

### Sensilla campaniformia (S.Ca)

These sensilla are also present on the distal end of fourth segment of maxillary palp (Fig. 3). These organs appear as a dome with a minute elliptical disc slightly inserted or sunken below the level of the integument and function as stress sense organs (mechano-sensilla).

### Sensilla squamiformia (S.Sq)

They are in the form of slender scales and are usually present among the scales of the maxillary palp (Figs. 1, 3, 4). These sensilla may be considered as mechanical in function where it may perceive stress in the cuticle resulting from mechanical deformation^18^.

### Effects of gamma radiation on the mouthparts and their sensilla

**Figure 5:**
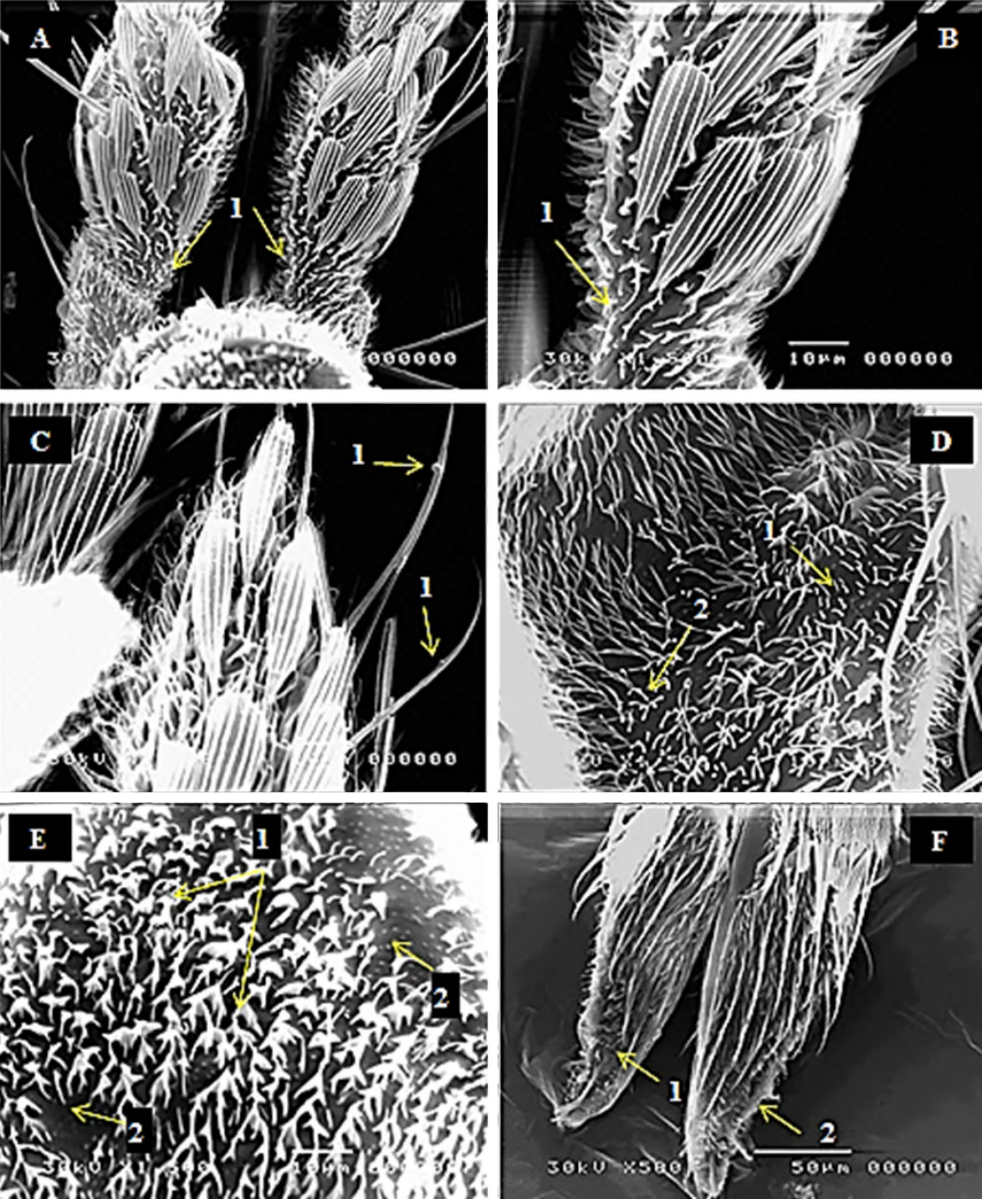
SEM photography of irradiated female mosquito, *Culex pipiens* as pupae with 20 Gy showing (**A-1**) falling of some sensilla squamiformia (S.Sq), (**B-1**) absent and disorganization of microtrichia (Mt) and (**C-1**) nodulation of sensilla chateica (S.Ch) at the maxillary palp segments. (**D-1&2**) decrease in density, twisted and dwarfed of sensilla trichodea (S.Tr), (**E-1**) malformation of disorientation of sensilla trichodea (S.Tr), and (**E-2**) empty pores at the clypeus. (**F-1&2**) showing changes in the normal shape of labial palps, lack of sensilla chateica (S.Ch) and sensilla trichodea (S.Tr).

It was noticed from the inspection by scanning electron microscope that dose of 20 Gy had a slight effect on the different types of the located sensilla comparing with the control. These effects showed many malformations in the maxillary palp segments as falling of some scales (sensilla squamiformia) at certain parts of the 1st segment leaving vacuoles. The number of microtrichia was decreased and became disorganized; also nodulation of sensilla chateica was noticed (Fig. 5A–C). Sensilla trichodea of clypeus showed some malformations such as twisted, dwarfed and disorientation. Also, these sensilla may fall leaving empty pores (Fig. 5D,E). The dose of 20 Gy caused changes in the normal shape of labial palps. On the other hand, lack of sensilla chateica and sensilla trichodea was noticed on this part (Fig. 5F).

**Figure 6:**
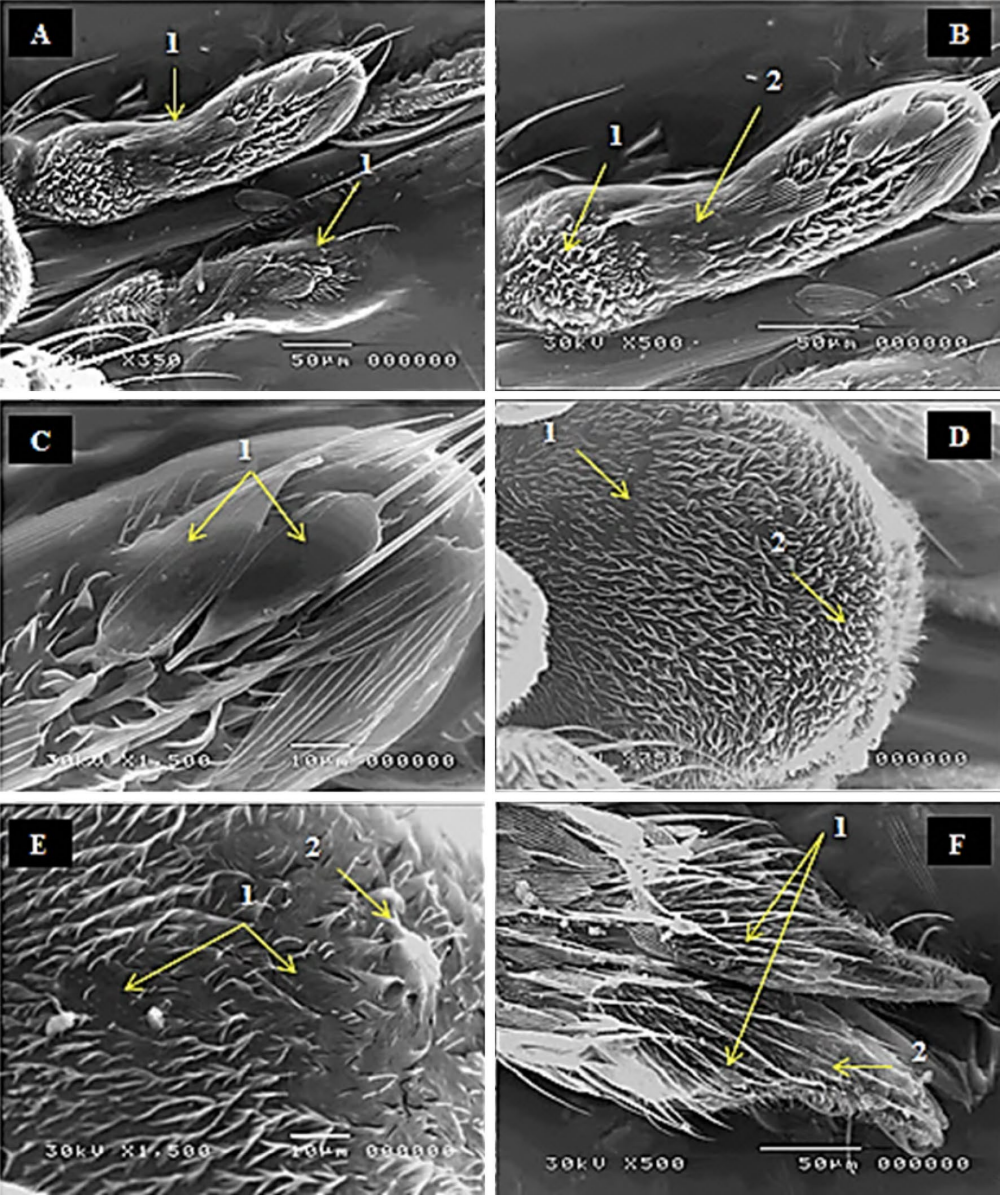
SEM photography of irradiated female mosquito, *Culex pipiens* as pupae with 40 Gy showing (**A-1**) liquefaction and changes in the normal shape of maxillary palp segments, (**B-1**) dwarfed and shrinkage of microtrichia (Mt), (**B-1**) falling of sensilla squamiformia (S.Sq). (**C-1**) absent and disorganization of sensilla squamiformia (S.Sq) at the distal end of maxillary palp. (**D-1&2**) lack and disorientation of sensilla trichodea (S.Tr) in some areas at the clypeus. (**E-1&2**) Showing liquefied or shrinkage of the clypeus at higher magnification. (**F-1**) broken of sensilla chateica (S.Ch), (**F-2**) scarcity sensilla trichodea (S.Tr) and sensilla basiconica (S.Ba) at the labial palps.

The different features of malformation increased progressively with increment of radiation dose. When the dose of radiation increased to 40 Gy, the maxillary palp segments were liquefied in some areas leaving undistinguished shape. The sensilla malformations were increased as observed at 40 Gy, where the number of dwarfed and shrinkage microtrichia was increased. At the distal end of maxillary palp, it observed that the sensilla squamiformia was absent and disorganization due to radiation effects (Fig. 6A–C). Trichodea sensilla the most sensitive sensilla found in clypeus became in collected form or bundles as a result of disorientation. Moreover, the surface of clypeus part became more liquefied or shrinkage (Fig. 6D,E). At the labial palps, the microscopic examination showed broken of sensilla chateica and scarcity of both sensilla trichodea and sensilla basiconica (Fig. 6F).

**Figure 7:**
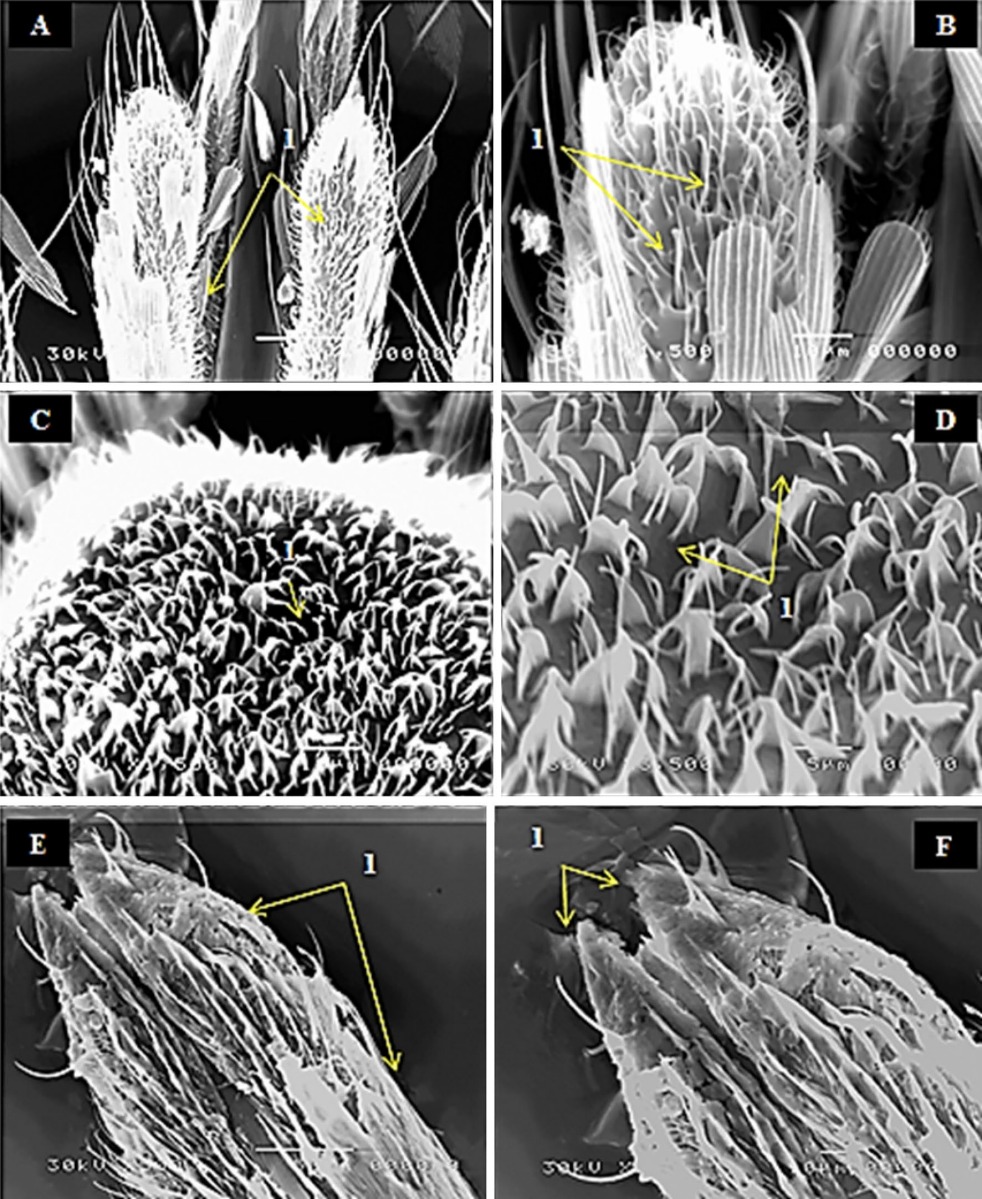
SEM photography of irradiated female mosquito, *Culex pipiens* as pupae with 80 Gy (**A-1**) showing falling of most sensilla squamiformia (S.Sq), (**B-1**) dwarfing and shrinkage of capitate peg sensilla (C.Ps) at the distal end of maxillary palp. (**C-1**) clumping and sticking of sensilla trichodea (S.Tr), (**D-1**) higher magnification of clypeus shows changes in the normal shape of sensilla trichodea (S.Tr). (**E-1**) showing destroy of most sensilla, falling of sensilla chateica (S.Ch), disorganization and loss of labial palps shape. (**F-1**) tear of the labial palps tip.

Treatment of 80 Gy has more malformation features than the previous treatment. Most of sensilla squamiformia was fallen along the maxillary palp segments leaving wide area of pores instead of them. The capitate peg sensilla became dwarfing and shrinkage at the distal end of maxillary palp (Fig. 7A,B). Trichodea sensilla at clypeus part had adequate number and these different types of trichodea bended at the basal end or at the distal end or twisted at their mid and may swell at their base, and they, also, may be sticking together forming bundles (Fig. 7C,D). At the highest dose 80 Gy, the different types of sensilla as trichoid, basiconic and chateica were most affected in the labial palps. In general, the labial palps shape became abnormal and in its tip had many tears (Fig. 7E,F).

## Discussion

Mosquitoes basically have two sets of olfaction organs or noses: antennae and maxillary palps that are covered by specialized sensory hairs called sensilla, which usually house two to three olfactory receptor neurons^19^. The obtained results indicated that there were six different sensilla identified along the mouthparts of female’s *Culex pipiens*. All sensilla observed, in this study could be displayed external morphologies similar to those displayed by the previous studies performed on antenna and mouthparts of female’s mosquitos. These sensilla play an important role in perception of thermal, chemical and mechanical stimuli^20^. Mosquitoes use an olfactory cue for nectar feeding, host finding, and oviposition^19^. In general, five types of stimuli are used by mosquitoes to locate hosts, namely visual cues, water vapours, heat, CO_2_ and body odour. The respective sensilla responding to these stimuli would be the compound eyes, grooved pegs, sensilla coeloconica, capitate pegs and sensilla trichoidea^21^. Once a female mosquito has landed the texture and perhaps taste of the host’s surface would be perceived first by the tactile setae and contact chemosensilla on the tarsi and subsequently labellum sensilla. Labial sensilla probably respond to cues in the blood during probing^21^.

The obtained results showed that the malformations in the mouth parts sensilla induced from exposing of female mosquito *Cx. pipiens* to the dose of 20 Gy was few, while the higher dose levels (40 and 80 Gy) had many harmful effects doses in the mouth parts sensilla. The percentage of malformations increased with the increase of gamma dose and this may affect feeding behavior. Irradiation with gamma rays on *Spodoptera littoralis* antennal sensilla showed that the trichoid sensilla became low in number and also showed the loss of the central pegs from some coeloconic sensilla. With the increase of doses some spines of the coeloconic were knobbed, plus point, the central pegs were lost^22^. Furthermore, Hazaa^13^ found that there are no malformations on squamiform or coeloconic sensilla by low doses of gamma rays but the trichoid sensilla showed swelling of their base on *S. littoralis* or slight warping or that they twisted together with loss in number. With increase of radiation dose, the malformation of sensilla increased as the trichoid sensilla disoriented and collected forming bundles of sensilla and the terminal parts nodulations and the basiconic shrank in many areas. At the high doses (150 Gy) the density of trichod sensilla became lower than in control at first generation to *Galleria mellonella* male^23^.

Hussien et al.^24^ stated that substerilizing doses of gamma radiation (5–12 Krad) reduced the density of trichoid sensilla and caused malformation to some of them in the antenna of the male moth of black cutworm, *Agrotis ipsilon*. Mahmoud and Gabarty^25^ showed that doses of 15 and 20 Gy have affected most types of males Red palm weevil, *Rhynchophorous ferrugineus* proboscis sensilla.

In general, the changes which occurred on the female mosquito mouth parts and their associated sensilla due to the doses of gamma radiation (20, 40 and 80 Gy) are in the form of shrunken, curved of sensilla into different directions, dwarfed and irregular in shape. As these sensilla are mechanosensitive, chemosensitive and olfactory in function, irradiation seemed to make insect fail in finding their niche or fail in courtship behavior^13^. El-Akhdar and Afia^26^ observed abnormalities in the antennal sensilla of *Bactrocera zonata* (saunders) treated with 90 Gy in full grown pupae and consequently affected the main role of the antennae of the released flies in determining the location of the host plant for feeding, oviposition, courtship, mating activity and mating behavior*.* The maxillary palp segments were liquefied in some areas at 40 Gy also, capitate peg sensilla which extremely sensitive to CO_2_ became dwarfing and shrinkage at the distal end of maxillary palp at 80 Gy and thus may lose his function in identifying the host.

Many authors studied the effect of gamma radiation on the sense organs in other insects; Ibrahim et al.^16^ on the antenna of *Culex pipiens* female, they recorded many morphological changes to the antennal segments and their associated sensilla due to the doses of gamma radiation (20, 40 and 80 Gy) such as shrinkage, curved of sensilla into different directions, irregularity in shape, swollen in some parts and some sensilla gathered into dense collections. These changes were obviously demonstrated at 40 Gy and 80 Gy doses whereas little malformations were observed when the pupae irradiated with 20 Gy dose. The antenna has lost most of different types of sensilla and some of its segments became transparent with high and moderate doses. Also, Sawires and Elbassiouny^27^ on *Rhyzopertha dominica* and El Degwi and Zarhan^28^ on *Bactrocera zonata.*

Different types of cuticular sensory receptors occur on various areas of the *Culex pipiens* female mouth parts to discriminate complex chemical and mechanical stimuli that are produced by the host. The present work describes various types of sensilla on mouth parts with distinct morphological characters and morphometric features. Sensilla on proboscis may respond to signals in blood through penetration^29^. The most abundant sensilla detected in the present investigation was sensillae trichoidea especially on clypeus and labial palps, with other kinds sensilla like sensilla chaetica, sensilla basiconica, capitate peg sensilla, sensilla campaniformia and sensilla squamiformia on the mouth parts of female mosquito, *Cx. pipiens*. Similar sensilla were fall under the family Culicidae^20^ such as, *Aedes aegypti*, *Ae. atropalpus*, *Ae. epactius*, and *Cx. pipiens*^30^, Aedes and Anopheles spp.^8^, *Ae. albopictus*^7^, Culex spp.^10^ *and Cx. pipiens*^16^. Sensilla trichodea has been described in different insects as having putative mechanoreceptive functions, such as the perception of mechanosensory stimuli^31^. These bristles are innervated by a single sensory neuron, ending with the typical tubular body, attached to the base of the hair shaft. This indicates a mechanosensory function^32^.

The maxillary palps consist of four segments, and contained other sensory structures such as, capitate peg sensilla, sensillae campaniformia, sensillae basiconica and certain non-innervated structures like cuticular projections, scales and microtrichia. These observations agreed with an earlier publication by McIver and Hudson; McIver and Siemicki, and McIver^20,33,34^ for other mosquito species and Seenivasagan et al.^7^ for *Ae. albopictus*. Kellogg^35^ reported that capitate pegs respond to acetone, amyl-acetate and n-heptane. Grant et al.^36^ studied the electrophysiology of peg sensilla on maxillary palps of *A. aegypti*, they found that these sensilla extremely sensitive to CO_2_. The importance of CO_2_ as a potential source of host/habitat cue in the sensory ecology of mosquitoes is highlighted by the fact that CO_2_ is the only odorant that consistently increased capture rates of many mosquito species^37^; peg sensilla are found in both sexes of culicine^38,39^ and anopheline mosquitoes^34^. From the results of the microscopic examination, it was found that the sensillae campaniform was observed on the distal end of fourth segment of maxillary palp, which consists of a domed cap that is hinged to the surrounding ring of raised cuticle. Similar structure was reported in *Anopheles stephensi*^40^. The sensilla campaniformia in insects are stimulated by passive mechanical deformations of the cuticle which can either be brought about by external forces or by self-produced movements^41^. Also, Day^42^ described a paired sense organ found in the head of adult mosquitoes, whose structure prompts the suggestion that it might be involved in the detection of changes in pressure. Lu et al.^43^ and Bohbot et al.^9^ found three innervated sensory organs on maxillary palp, capitate sensillae basiconica, which were porous hairs with 3 neurons of chemosensory sensitive to CO2, octenol and human skin odorants, and sensillae campaniformia and chaetica were mechanoreceptors holding one sensory neuron.

The labial palps of mosquitoes were considered by many workers as important in serving the fascicle during piercing and sucking also Jones and Pilitt^44^ found that removal of the labella results in the failure of mosquitoes to penetrate the skin, thus showing the importance of the labella during piercing. The types of the sensilla found on the labial palps of female *Cx. Pipiens* were three size of smooth sensilla chateica at the tip of labellum, sensilla trichodea, sensilla basiconica and sensilla squamiformia. Our observations were supported by earlier findings of Hill and Smith^45^; Amer and Mehlhorn^8^ in *Ae. aegypti* and *An. stephensi*.

Exposure to ionizing radiation is the preferred method for making insects reproductively sterile for integrated pest management (IPM) programs that integrate sterile insect technology (SIT). Therefore, the objective of this study is whether there is any conflict between the use of gamma radiation in the control of *Cx. pipiens* and its role in increasing the damage from irradiated females and their reach to the host and what causes other damages such as biting and blood sucking as well as the transmission of diseases compared to non- irradiated insects. Also, the selected radiation doses are sterile doses for *Cx. pipiens* according to the obtained results from Hassan et al.^46^ thus the irradiated females will not lay eggs and will not cause an increase in the mosquito population if released.

## Conclusion

By using scanning electron microscopy, the conclusion obtained results that gamma radiation cause malformation in the olfactory and gustatory sensilla on the mouthparts of female mosquito. Accordingly, if we release irradiated mosquito (males and females) due to the difficulty of distinguishing between them at pupal stage, females will not be able to reach their host in order to obtain the blood meal necessary for laying eggs. So female mosquito will not be able to reproduce given new individuals and we can protect ourselves from mosquito-borne diseases.

## Data Availability

All data generated or analyzed during this study are included in this published article.
